# Effect of cortex inactivation on spontaneous activity of cells in perigeniculate and dorsal lateral geniculate nuclei

**DOI:** 10.1186/1471-2202-14-S1-P418

**Published:** 2013-07-08

**Authors:** Jacek Rogala, Wioletta J Waleszczyk, Andrzej Wróbel, Daniel K Wójcik

**Affiliations:** 1 Department of Neurophysiology, Nencki Institute of Experimental Biology, Warszawa, 02-093, Poland

## 

The cortical influence on spontaneous activity of single neurons in the dorsal lateral geniculate (LGN) and perigeniculate (PGN) nuclei were investigated in awake cats by means of reversible cooling of cortical areas 17 and 18 [1]. We analyzed subtle changes of the statistical properties of spike trains. To understand their nature we investigated statistics of firing rate (FR), mean inter-spike interval (ISI), bursting rate (BR), mean intra-burst inter-spike interval (IBISI), mean number of spikes per burst (SPB) and mean burst duration (BD)[2,3].

These results indicate that recurrent inhibition from PGN may play a homeostatic role in the cortico-thalamic loop by restricting thalamic oscillations within their natural functional range [4]. The buffering role of PGN may explain why the massive, direct cortical projection to LGN neurons induces only small, highly-tuned effects during spontaneous activity.

**Figure 1:**
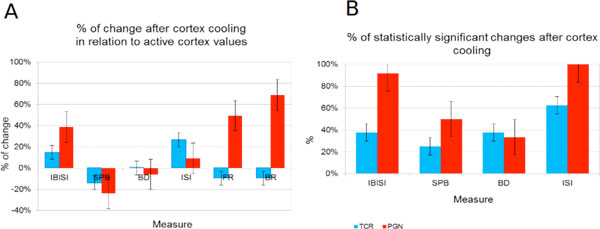
**Effect of cortex cooling on activity of PGN and LGN cells**. A. Percent of change in relation to situation when the cortex was active. B. Percent of significant differences (p < 0.05). IBISI - intra burst ISI, SPB - spikes per burst, BD - burst duration, ISI - mean ISI, FR - firing rate, BR - bursting rate.

The BR, FR and IBISI measures of spontaneous activity of PGN cells as a result of cortex cooling increased more than 50% of their original values and more than 20% for SPB while the changes of LGN activity were below 20% for all measures except ISI, which was the only case where the changes were larger in LGN than in the PGN cells (Figure 1A). The changes of values taken by all the measures used to quantify activity of LGN (except mean ISI) were statistically significant for less than 30% of investigated neurons, in the case of PGN all the changes except for BD were significant for more than 40% of neurons (Figure 1B).
